# Species-specific model to predict amphibian metamorphosis

**DOI:** 10.1038/s41598-023-43639-0

**Published:** 2023-10-02

**Authors:** Noriko Iwai, Yuuya Tachiki

**Affiliations:** 1 Department of Environment Conservation, Tokyo University of Agriculture and Technology, 3-5-8 Saiwai-cho, Fuchu, Tokyo 183-0054 Japan; 2 Department of Biological Sciences, Tokyo Metropolitan University, 1-1, Minami-Osawa, Hachioji, Tokyo 192-0397 Japan

**Keywords:** Ecology, Evolution, Zoology

## Abstract

Exploring the timing of life-history transitions has been a pivotal focus in the field of evolutionary ecology. Studies on amphibian metamorphosis are well suited to investigate this aspect. We propose a species-specific model to predict the optimal metamorphosis point for frog individuals with different larval growth trajectories. Because overall fitness will be determined throughout both aquatic and terrestrial stages, we included growth and survival rates of aquatic and terrestrial stages in the fitness equation. Then we conducted a rearing experiment on a brown frog, *Rana ornativentris*, as an example to obtain the size at metamorphosis, larval period, and larval growth trajectory. Based on these results, we determined the model's parameters to fit the actual metamorphosis patterns. Because the parameters are supposed to be evolutionarily maintained, our data-driven approach enabled obtaining fundamental ecological information (evolutionally-based life-history parameters) of the target species. Comparing the parameters among species will allow us to understand the mechanisms in determining life-history transition more deeply.

## Introduction

How animals determine the timing of life-history transitions has been a fundamental question in evolutionary ecology^1–3^. Hatching, emergence, metamorphosis, and maturation occur at some point in life, and transitioning at the right time according to the species and individual situation is essential to obtain high fitness^4, 5^. Thus, species are expected to have evolved fine-tuned life-history strategies to adjust the timing optimally. Several theoretical, mathematical, and experimental studies have focused on this aspect^3, 6–9^, with size at and time to developmental milestones considered as effective predictors for future fitness^10, 11^.

Amphibian metamorphosis is the best-suited field to study this phenomenon. Most amphibians have complex life cycles with an aquatic larval and terrestrial adult stage^12^. Many studies have tried to explain how amphibians determine the timing of metamorphosis^13–16^. Researchers have shown that, in many cases, metamorphosis should occur at larger sizes because a larger size at metamorphosis often leads to greater fitness through higher survival rates and faster growth rates in terrestrial stages^17–19^. However, larvae need more time to reach a larger size and must stay in the aquatic environment for longer. This is sometimes unfavorable because larvae will be exposed to desiccation and predation risks in the aquatic environment^20^. Thus, larvae struggle to solve the tradeoff between becoming early-small vs. late-large metamorphs.

A well-known theory to explain amphibian metamorphosis has been advocated by Wilbur and Collins^6^: there are minimum and maximum body sizes, metamorphosis can occur in between, and the larvae determine the size at metamorphosis depending on their recent growth history. For example, slowly growing larvae will metamorphose once they reach the minimum size threshold because postponing the larval period will lead to a higher aquatic mortality rate that does not offset the benefit of being larger^21^. This conceptual model explains how larvae of a certain species time the life-history transition under different environmental conditions^7, 22, 23^. Several studies have tried to verify the model with rearing experiments or field surveys, resulting in several different models^7, 13, 23^. However, the limitations of the Willbur–Collins model have not yet been overcome. For example, the model is only descriptive and does not explain why the larvae change their strategy at a certain growth rate. The model is simple and thus could be applied to most species; however, this is at the expense of specificity and does not enable us to determine how and why the responses would differ among species.

Another limitation of the Willbur–Collins model is that it only considers the aquatic stage for the decision of metamorphosis. However, metamorphosed individuals grow and survive in the terrestrial stage afterward, and their overall fitness will be determined throughout the aquatic and terrestrial stages. Werner^24^ proposed an elegant graphical model that incorporated this aspect. He considered growth rates (a function of the body size *s*; $g\left(s\right)$) and mortality rates ($\mu \left(s\right)$) of aquatic and terrestrial life stages and showed that minimizing $\mu \left(s\right)/g\left(s\right)$ throughout life determines optimal transition points for each species. Although this model was originally proposed to explain different metamorphosing strategies between species, it can also explain differences within species. Thus, this model successfully showed the optimal metamorphosing point with definite reason while explaining species-specific metamorphosing strategies. This model, however, is difficult to verify because it is challenging to obtain $\mu \left(s\right)$ and $g\left(s\right)$ of both aquatic and terrestrial life stages in the field. Due to the lack of data, the model cannot show where the specific optimal metamorphosing point is for each individual of each species.

We aimed to develop a species-specific model that incorporated the optimal metamorphosing point for individuals with different growth trajectories and aquatic and terrestrial stages' growth and survival rates. Subsequently, the model was fitted to the data of a rearing experiment with a brown frog, *Rana ornativentris* to obtain parameters through optimization approach*.* Because the metamorphosing point is supposed to be evolutionarily maintained, the estimated parameters provided basic ecological information about the target species. Our approach allowed us to estimate the specific optimal metamorphosing point for any individual of the focal species under different growth history, and to provide insights into the reasons behind such decision.

## Results

In the tadpole rearing experiment, one individual had not metamorphosed by day 232 when the experiment was terminated. Seven tadpoles died before the metamorphosis. We excluded data from five individuals in the treatment group with the least amount of food because they metamorphosed on days 93–144, which is exceptionally late and inconceivable in the field. Therefore, 29 successful growth curves were obtained. Size at metamorphosis ($actS$) ranged from 333.0 to 884.7 mg with a mean of 533.7 ± 154.1 (SD, N = 29) mg. The larval period ranged from 41 to 80 days with a mean of 51.4 ± 8.6 (SD) days.

**Figure 1:**
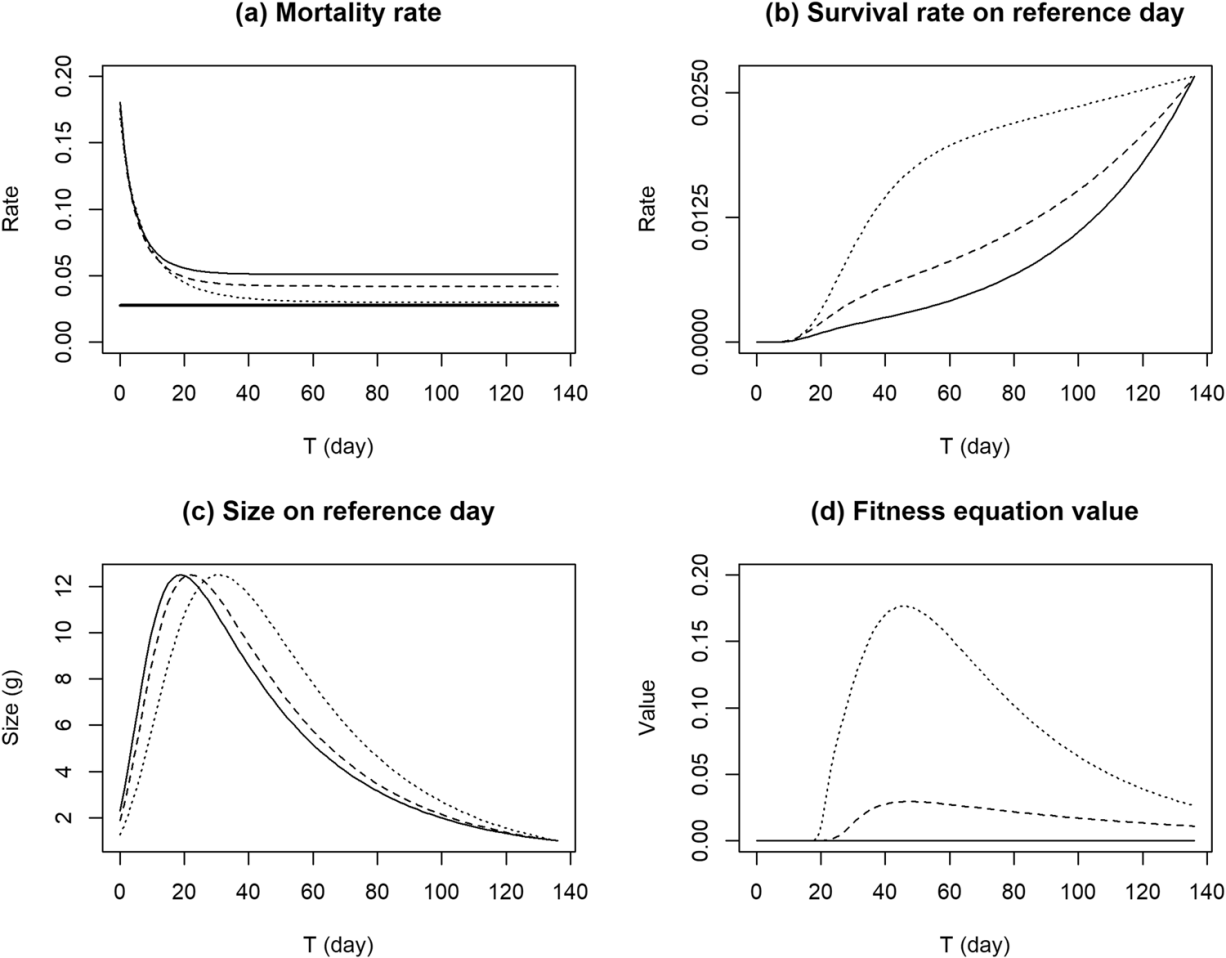
Behaviors of fitness elements according to larval period, $$T$$ (day). (**a**) mortality rate of aquatic and terrestrial stages; (**b**) survival rate on reference day; (**c**) size on reference day; (**d**) fitness equation value. The dotted line shows the value for an individual with the largest size at metamorphosis ($$actS=884.7, actT=47)$$, a broken line for one with median size at metamorphosis ($$actS=486.5, actT=48)$$, and a solid line for one with the smallest size at metamorphosis ($$actS=333.0, actT=57)$$. The aquatic mortality rate was identical for all the individuals ($${\mu }_{\mathrm{A}}$$: 0.027), thus shown as a bold line in (**a**).

The Gompertz equation fitted well with the growth trajectories with a high correlation coefficient (all > 0.95). $U$ ranged from 333.4 to 1034.4, *G* from 0.024 to 0.060, and *I* from 5.54 to 19.2 (Appendix 1). The genetic algorithm revealed that the smallest deviation between optimal and actual metamorphosis points was achieved with *Z*: 455.4, *a*: 21.4, *r*: 0.028, ${\mu }_{\mathrm{A}}$: 0.027, *M*: 0.87, and *b*: 0.49. As the size at metamorphosis ($actS$) ranged from 333.0 to 884.7 mg, the terrestrial mortality rate (${\mu }_{\mathrm{T}}$) was from 0.032 to 0.051. This was always higher than the aquatic mortality rate (${\mu }_{\mathrm{A}}$: 0.027, Fig. 1a), meaning that the tadpoles that stayed in the aquatic environment longer would have a higher survival rate on the reference day (Fig. 1b). Tadpoles still metamorphosed before reference day because the growth plateaus in the aquatic stage and, thus, large tadpoles would not be expected on the reference day if they stayed in the aquatic environment too long (Fig. 1c). The combination of mortality and growth rates of aquatic and terrestrial environments generated the concave fitness curve along the larval period (Fig. 1d), and thus gave us the optimal point for metamorphosis.

**Figure 2:**
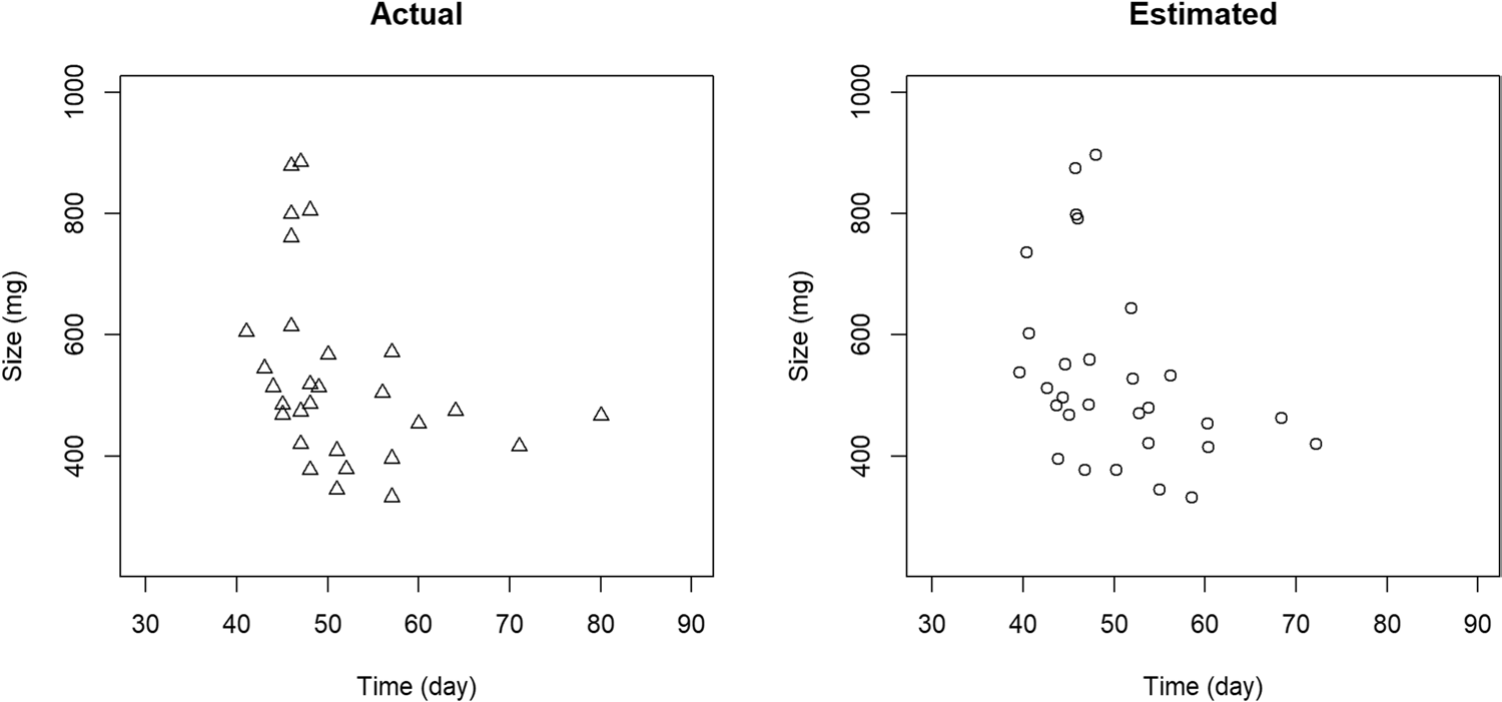
Larval period and size at metamorphosis of 29 *Rana ornativentris* tadpoles. Left: actual data from the rearing experiment. Tadpoles were reared with different amounts of food to induce variations in growth rates. Right: estimated data using fitness equation.

The estimated optimal and actual metamorphosing points were scattered closely (Fig. 2). Growth trajectories through aquatic to terrestrial stages for each individual were realistic, and the size on reference day ranged from 2.2 to 10.6 g (Appendix 2).

## Discussion

We obtained a species-specific model explaining the decisions in the life-history transition between two different ecosystems. Our approach is novel because we adopted a fitness equation and calculated an exact “optimal point” on each growth trajectory. Assuming that the target species have evolved to realize the optimal point according to their growth trajectories, we back-calculated the parameters in the fitness equation (Eq. 10) based on rearing experiment data. Thus, our data-driven approach allowed us to obtain evolutionally-based life-history parameters rather than instant parameters specific to each experiment (e.g., place, year, and season).

Life-history parameters estimated by our approach were reasonable, supporting the validity of our approach. For example, the obtained aquatic mortality rate gives a cumulative survival rate during the larval period of 0.12–0.34, depending on the length of the period. Other studies showed that survival during the aquatic period in nature was 0.23 for *Phasmahyla jandaia*^25^ and 0.625 for *Rana sylvatica*^26^. Seigel^26^ noted that this high value was probably because of the lack of predators and short larval period (< 2 months). The value in our study is not influenced by this and shows an evolutionally-based value for the species; around one-third of the *R. ornativentris* hatchlings would be expected to leave the pond as metamorphs.

The obtained terrestrial mortality rate resulted in a survival rate from metamorphosis to reference day (end of summer) of 0.014–0.088. A mark-recapture study showed that the first-year juvenile survival was around 0.2–0.4 in *Pelobates fuscus*^27^ and 0.018–0.332 (with considerable variation) in *R. sylvatica*^28^. The terrestrial survival rate in our study was at the lower end of the range of these previous studies. However, the early period of froglets (soon after metamorphosis) will show a low survival rate. Survival during the first two weeks was sometimes less than 0.5 in *Rana lessonae* and *Rana esculenta*^19^, equivalent to ${\mu }_{\mathrm{T}}$ = 0.050. Our study estimated terrestrial survival rate for the first 8–14 weeks, and it would be reasonable to have a lower value than the first-year value. Alternatively, the high survival rate in *P. fuscus* may be due to their fossorial behavior^29^. *R. ornativentris* is ecologically more similar to *R. sylvatica* as both are brown frogs with the same size range and may thus have similar survival rates.

Growth trajectories across two stages (larvae and adults) were also realistic. To investigate this, we drew growth trajectories throughout aquatic and terrestrial stages until the reference day (Appendix 2). According to the growth profile, the size at the end of the first summer was 2.2–10.6 g (4.7–13.9 times growth in 8–14 weeks). The study on *Rana dybowskii* showed 7–8 times growth in body weight during the first 18 weeks in captivity^30^, which is equivalent to our data. The adult size of *R. ornativentris* was 8–16 g for males and 7–21 g for females (both N = 8; Iwai, unpublished data), probably gained over 1–2 years. Considering this, the size at the end of the first summer reflected reality, supporting the validity of our approach.

Establishing life-history parameters in natural conditions is often difficult without long-term intensive fieldwork^27, 28^. Considering that the estimated life-history parameter values were realistic, as we mentioned above, our approach will allow obtaining fundamental species information for many species with less effort than before. Future studies comparing such information among species with different life history traits will be interesting.

With the evolutionally-based life-history parameters, our model can also explain the extreme life-history strategies, such as neoteny and direct development. For example, parameters of sufficiently small ${\mu }_{\mathrm{A}}$, large ${\mu }_{\mathrm{T}}$, small *r*, and/or large *b*, can give us $optT=R$ for any growth trajectory, corresponding to neoteny. Alternatively, parameters of sufficiently large ${\mu }_{\mathrm{A}}$, small ${\mu }_{\mathrm{T}}$, large *r*, and/or small *b*, can give us $optT=0$, that is, direct development. Neoteny occurs under a favorable, stable aquatic environment combined with a harsh terrestrial environment^31^, while direct development proceeded in deteriorating larval habitats with a highly favorable adult habitat^32^. Unfortunately, these previous studies have been restricted to descriptive or modeling prediction without actual data. Our data-driven approach will enable an explanation of the extreme strategies by using life-history parameters. Rearing experiments on those species with extreme strategies will be interesting to verify the above idea in the future.

Another benefit of our novel approach was that it freed the model from minimum and maximum size at metamorphosis while containing its concepts more efficiently. Previous models have often considered those thresholds following Wilbur and Collins^6^, but their definition of the thresholds did not fit reality. In terms of the maximum size, they stated that “the body size that takes so long to achieve that the probability of drying of the pond, or the onset of winter, is so great that natural selection has favored the initiation of metamorphosis rather than the risk of further growth in the aquatic habitat”. The period each larva takes to achieve the maximum size, however, should differ depending on the growth rate, and thus the maximum size, as Wilbur and Collins^6^ determined, should not be a constant for a species. Rather, it should change according to the individual growth rate. In our model, the benefit of being large and the cost of taking longer to become large are both considered mathematically, which inevitably gives optimal size without explicitly including “maximum size”.

**Figure 3:**
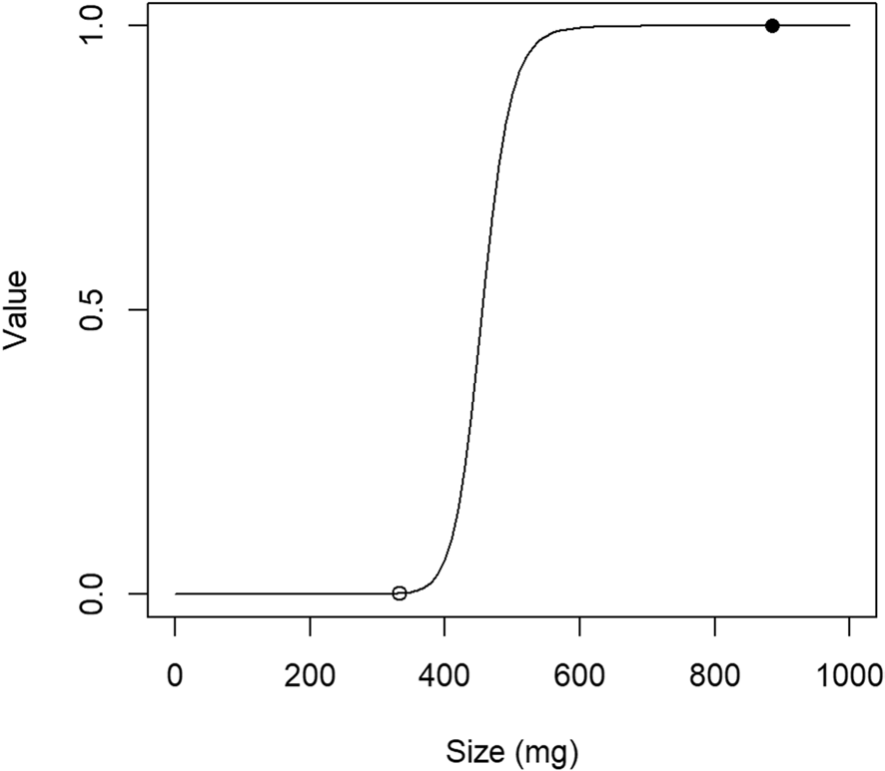
Behavior of the value of Eq. (3) according to the size at metamorphosis ($S$). The open circle shows the observed minimum size at metamorphosis ($actS=333.0$), and the filled circle shows the observed maximum size at metamorphosis ($actS=884.7$).

Similarly, setting a constant for the minimum size at metamorphosis was inappropriate because there can be a gradual response over the threshold. In Wilbur and Collins^6^, minimum size was stated as the “lower limit of body size, determined by threshold responses to the endocrinological mechanisms that initiate metamorphosis” and thus a constant threshold for a species. In this study, this hypothesis was incorporated in our model more effectively as Eq. (3), showing a shift of fitness contribution around a certain size with a value from 0 to 1. For example, according to the obtained model for *R. ornativentris*, the Eq. (3) was equal to 0.0012 at the smallest observed size at metamorphosis (333.0 mg, Fig. 3). Individuals that metamorphosed at a smaller size than this would have a value smaller than 0.0012 for Eq. (3), meaning they are not worthy of metamorphosis (fitness equation value is almost 0). This is similar to having a threshold, except that the response is gradual instead of 0/1 on the threshold. This “gradational threshold” can reflect diverse steepness in the responses among species. If *a* is small, the contribution to fitness does not change dramatically but shows a mild increase with increasing body size $S$. If *a* is large, Eq. (3) works more like a “threshold”, where individuals under a certain value are close to 0 while those that exceeded the threshold obtain a value close to 1. A small *a* may belong to those species in which small size excess over the “threshold” is not very important for fitness, such as those whose size at metamorphosis is inherently large^10^. However, a large *a* may be observed in the species whose size at metamorphosis is inherently small, and thus small decreases in size may be critical. Considering different responses among species toward minimum size will help understand how life history transition strategies are associated with the species’ characteristics.

We presented a novel approach to understanding the mechanism of decisions in life-history transitions. Our approach is new because it gave us the fitness equation that is effectively reflecting species-specific strategy, which enabled us to obtain fundamental life-history parameters such as mortality and growth rates of multiple life stages. With this fitness equation and growth trajectories, we can now estimate the exact optimal metamorphosing point for each individual of the focal species. Our data-driven approach can be adapted to any group level, such as the population, if the group is expected to have evolved its own strategy. Comparing fitness equations among groups will allow us to understand the mechanisms of the evolution of complex life history more deeply.

Our study provides a base for future studies. Modified versions of our model can be applicable if it suits the target species. For example, size-specific growth and/or mortality rates can be included in aquatic and terrestrial stages. Growth and mortality rates can be different between sexes. Terrestrial growth can be asymptotic later in the season and/or as growth proceeds. Factors other than growth and mortality can be included, such as temperature. Environmental conditions can be changed during the larval period. The presumptions we made will depend on what feature is important for the target species, and how simple we want the model to be.

## Methods

### General description

**Figure 4:**
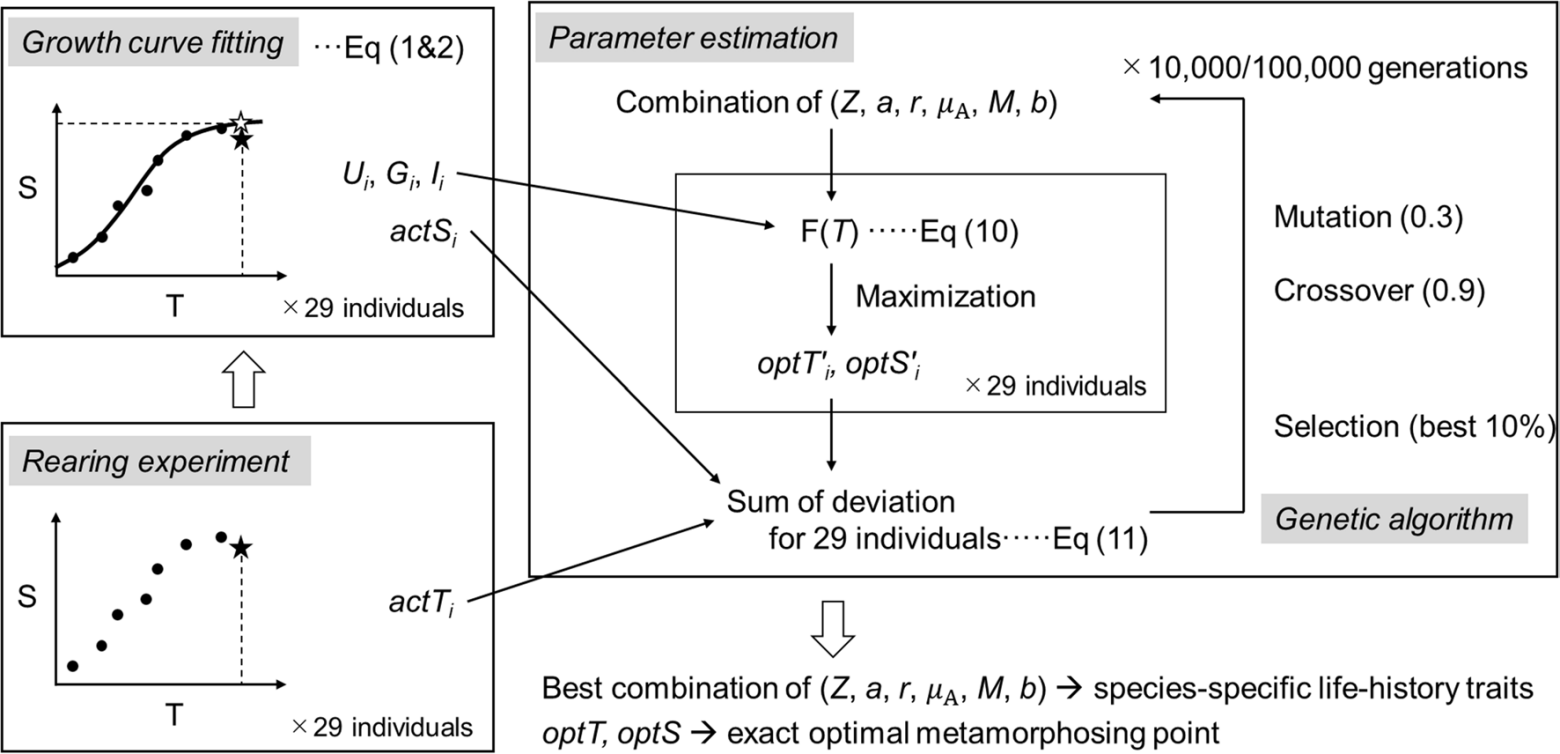
Scheme of this study.

We reared *Rana ornativentris* tadpoles with different food treatments to obtain diverse growth trajectories and consequential actual metamorphosis points. Next, we fitted the Gompertz growth equation for the individual growth trajectories. We then constructed a fitness equation that includes parameters reflecting the growth and survival of both aquatic and terrestrial stages. Under a certain combination of fitness equation parameters, we can calculate the optimal metamorphosis points according to the individual growth curve. Assuming that the reared tadpoles showed optimal responses, we determined parameters by minimizing the sum of deviations between actual (obtained in rearing experiments) and estimated optimal metamorphosis points through a genetic algorithm. The scheme of this study is shown in Fig. 4 and detailed methods are provided below.

### Rearing experiments

*Rana ornativentris* tadpoles were reared in a laboratory to obtain data on individual growth curves and consequential actual size and age at metamorphosis. Variations in aquatic growth rates were created by varying the amount of available food.

Two egg masses were collected from the field on April 9, 2017 and incubated until they hatched. When tadpoles reached Gosner^33^’s stage 25 on April 17, they were individually placed in a plastic container (129-mm diameter, 72-mm height) with 300 mL of aged tap water (day 0). Individuals were randomly assigned to one of the six food treatment groups (shown below) with seven individuals per group. In total, six or nine individuals that hatched from each egg mass were photographed with a scale to estimate the initial body weight using a formula for the relationship between snout-vent-length (SVL) and body weight obtained from measuring 468 *O. splendida* tadpoles in another study (W(mg) = 206 × SVL(cm)^2.85^). Containers were incubated (MIR-254-PJ or MIR-554-PJ, Panasonic, Japan) at a constant temperature (typical of the species’ natural habitats, 18 °C) and a 12:12 h light:dark cycle.

Commercial fish food (Hikari-haiga, Kyorin, Japan) was crushed to a powder, mixed with a 1% agar solution, and placed in a Petri dish of 84 mm diameter. Food pellets were bored using straws of different diameters, and a fixed amount of food (1.6, 2.5, 3.2, 4.1, 5.0 mg/day, and ad libitum) per individual was supplied every 3 or 4 days when the water was changed and the containers cleaned. The amount of food was adjusted to encompass a spectrum ranging from limited to abundant to capture a diverse range of growth rates. In the “ad libitum” treatment, we ensured there was always leftover food. Body weight was measured once every 3–21 days.

Metamorphosis was defined as the emergence of the first forelimb and was inspected daily. The day of the observed metamorphosis was determined as the actual metamorphosis time ($$actT)$$. The metamorphosed individuals were kept in the same container with a small amount of water until complete tail resorption. Because the body weight of metamorphosing individuals decreased greatly until tail resorption, the measured body weight of metamorphosed individuals was not used. Instead, the actual size at metamorphosis ($$actS$$) was calculated by substituting the age at metamorphosis in the individual growth curve (see below).

### Growth curve and actual metamorphosis point (${\varvec{a}}{\varvec{c}}{\varvec{t}}{\varvec{T}}$, ${\varvec{a}}{\varvec{c}}{\varvec{t}}{\varvec{S}}$)

We assumed that the growth patterns of tadpoles followed the Gompertz equation. Following Tjørve and Tjørve^34^, the unified Gompertz equation was used:

1 $$S(t)=U\mathrm{exp}\left(-\mathrm{exp}\left(-eG\left(t-I\right)\right)\right)$$

where *S*(*t*) is the size at age *t*, *U* represents the upper asymptote, *G* represents the maximum relative growth rate at inflection, and *I* represents the inflection age.

Age (day) and body weight (mg) data obtained from the rearing experiments were substituted into Eq. (1) for each individual. Late-stage tadpoles close to metamorphosis often show decreasing body weight, which should not be a part of the growth curve. Thus, we eliminated body weight data from seven days before metamorphosis if the weight was lower than the previously measured value. The best fitted *U*_*i*_, *G*_*i*_, and *I*_*i*_ were determined using the R-software ver. 4.1.2 (R Foundation for Statistical Computing, Vienna, Australia) and the “nls” function^35^ for each *i*th individual to minimize deviation. The actual size of *i*th individual at metamorphosis ($act{S}_{i}$) was then determined as the size on the actual date of metamorphosis ($act{T}_{i}$) for individual Gompertz-fit growth curves as follows:

2 $$act{S}_{i}={U}_{i}\mathrm{exp}\left(-\mathrm{exp}\left(-e{G}_{i}\left(act{T}_{i}-{I}_{i}\right)\right)\right)$$

### Fitness equation

The fitness equation was constructed from four terms related to aquatic growth, terrestrial growth, aquatic survival, and terrestrial survival. To incorporate the hypothesis that metamorphosis determines the distribution of growth and the survival period between aquatic and terrestrial stages, we set a reference day, $R$, assuming that the size on and survival until this day reflects the fitness. In this study, we used $R=136$ (31 August) assuming that exponential terrestrial growth can be expected until the end of the summer. Larvae were expected to metamorphose between days 0 and 136. If larvae stay in the aquatic stage for longer, they are exposed to aquatic growth and survival rates longer while spending less time in the terrestrial stage with less exposure to terrestrial growth and survival rates.

The consequence of aquatic growth can be regarded as size at metamorphosis ($$S$$). This size at metamorphosis can affect fitness through power-law scaling with a constant (*a*: $${S}^{a})$$. To incorporate the hypothesis of minimum size at metamorphosis^6^, we expressed this term as a hill equation with constant Z:

3 $$\frac{{S}^{a}}{{Z}^{a}+{S}^{a}}$$

This equation can represent a sudden steep increase (i.e., passing the minimum size) and sudden leveling off (i.e., close to the maximum size), with a value of 0.5 when Z = *S*.

The terrestrial growth is expressed as an exponential increase over the duration until the reference date $(R-T)$ with initial size ($S$) and constant growth rate (*r*):

4 $$S\mathrm{exp}\left(r\left(R-T\right)\right)$$

Aquatic survival is expressed as an exponential decrease with aquatic mortality rate (${\mu }_{\mathrm{A}}$) over the larval period ($T$):

5 $$\mathrm{exp}\left(-{\mu }_{\mathrm{A}}T\right)$$

Terrestrial survival is expressed as an exponential decrease with terrestrial mortality rate ${\mu }_{\mathrm{T}}$ over the time until the reference date $(R-T)$.

6 $$\mathrm{exp}\left(-{\mu }_{\mathrm{T}}\left(R-T\right)\right)$$

To consider the positive effect of size at metamorphosis on terrestrial survival, we assumed that ${\mu }_{\mathrm{T}}$ is the reverse proportion of the size at metamorphosis of power-law scaling. Then ${\mu }_{\mathrm{T}}$ is expressed with a constant $M$ and *b:*

7 $${\mu }_{\mathrm{T}}=\frac{M}{{S}^{b}}$$

Using terms (6) and (7), terrestrial survival is now expressed as:

8 $$\mathrm{exp}\left(-\frac{M}{{S}^{b}}\left(R-T\right)\right)$$

All the above terms will act synergistically; thus, the complete fitness function is given as the multiplication of (3), (4), (5), and (8):

9 $$F(S, T)=\frac{{S}^{a}}{{Z}^{a}+{S}^{a}}\times S\mathrm{exp}\left(r\left(R-T\right)\right)\times \mathrm{exp}\left(-{\mu }_{\mathrm{A}}T\right)\times \mathrm{exp}\left(-\frac{M}{{S}^{b}}\left(R-T\right)\right)$$

Because $S$ can be expressed by $T$ through Eq. (1), Eq. (9) is now expressed as an equation with a single variable (*T*) as:

10 $$F(T)=\frac{{(U\mathrm{exp}\left(-\mathrm{exp}\left(-eG\left(T-I\right)\right)\right))}^{a}}{{Z}^{a}+({U\mathrm{exp}\left(-\mathrm{exp}\left(-eG\left(T-I\right)\right)\right))}^{a}}\times U\mathrm{exp}\left(-\mathrm{exp}\left(-eG\left(T-I\right)\right)\right)\mathrm{exp}\left(r\left(R-T\right)\right)\times \mathrm{exp}\left(-{\mu }_{\mathrm{A}}T\right)\times \mathrm{exp}\left(-\frac{M}{{(U\mathrm{exp}\left(-\mathrm{exp}\left(-eG\left(T-I\right)\right)\right))}^{b}}\left(R-T\right)\right)$$

### Parameter estimation

To estimate parameters that reflect the actual metamorphosing points the best, we implemented a genetic algorithm using a “ga” function^36^ in software R. For a certain combination of six parameters (*Z*, *a*, *r*, $${\mu }_{\mathrm{A}}$$, *M*, *b*), optimal age at metamorphosis for *i*th individual ($$opt{T}_{i}$$) can be determined to maximize Eq. (10) with a limitation of $$opt{T}_{i}<R$$. Other parameters ($$U$$, $$G$$, and $$I$$) were previously determined for individuals with the actual growth curve (see above). Optimization was conducted using the “optimize” function in software R. With the $$opt{T}_{i}$$, the optimal size at metamorphosis ($$opt{S}_{i}$$) can be calculated through Eq. (1), and thus the optimal point at metamorphosis ($${optT}_{i}, {optS}_{i})$$ is determined for all the individuals. We then calculated the sum of deviation ($$SD$$) between optimal (opt$${T}_{i}, {optS}_{i})$$ and actual ($${actT}_{i}, {actS}_{i})$$ metamorphosis points:

11 $$SD=\sum_{i=1}^{N}\left(({optT}_{i}-{actT}_{i})^{2}+{\left({optS}_{i}-{actS}_{i}\right)}^{2}\right)$$

The genetic algorithm was run to minimize this sum of deviation (11) under different combinations of six parameters. The range of parameters were set as follows; *Z*: 0–2000, *a*: 1–100, *r*: 0–0.05, ${\mu }_{\mathrm{A}}$: 0.001–0.1, $M$: 0.001–100, *b*: 0–1. To set terrestrial mortality ($\frac{M}{{S}^{b}}$) within the same range as that for aquatic mortality (0.001–0.1), $-1000M+{250}^{b}<0$ and $10M-{900}^{b}<0$ were used as constraints considering the range of $S$ as 250–900.

The initial population size was set at 1000, the mutation probability was 0.3, and the crossover probability was 0.9. The top 10% of best fitness individuals survived each generation. The genetic algorithm was run 5 times for 10,000 generations and 10 times for 100,000 generations. The solution with the minimum deviation was chosen as the best.

### Ethical approval

Rearing experiment of tadpoles were performed in accordance with Animal Care Guidelines for Herpetology developed by the American Society of Ichthyologists and Herpetologists, and the committee of animal experiments at the Tokyo University of Agriculture and Technology. The study is reported in accordance with ARRIVE guidelines ([https://arriveguidelines.org).

### Supplementary Information

Supplementary Information.

## Data Availability

Datasets are available at: https://datadryad.org/stash/share/wCKKMs_eTkMQatr5jybWGP2RcDUrauT2-tmtzxbvo8c.
