# Supplementary material for: Species-specific model to predict amphibian metamorphosis
Source: Sci Rep. 2023 Oct 2;13:16503. doi: 10.1038/s41598-023-43639-0 (PMC10545764; doi:10.1038/s41598-023-43639-0)
Supplement: Supplementary file 1 — Supplementary Information. [file 41598_2023_43639_MOESM1_ESM.pdf]

Appendix 1. Parameters of Gompertz growth curve, actual and optimal larval period (*T*, day), and size at metamorphosis (*S*, mg) for 29 *Rana ornativentris* tadpoles.

ID	*U*	*G*	*I*	*actT*	*actS*	*optT*	*optS*
B1	379.6	0.058	6.3	52	379.3	46.8	379.0
B2	333.4	0.049	7.1	57	333.0	58.5	333.1
B3	346.5	0.051	6.9	51	345.7	55.0	346.1
B5	470.1	0.029	12.6	80	467.7	68.3	464.2
B6	396.8	0.060	5.5	57	396.7	43.8	396.0
B7	417.6	0.040	8.7	71	417.1	60.3	416.1
C1	379.0	0.054	6.8	48	378.0	50.2	378.3
C2	422.5	0.032	11.5	51	409.6	72.2	420.5
C3	424.0	0.046	8.3	47	420.7	53.7	422.6
C4	476.8	0.038	9.6	64	475.2	52.7	471.6
C6	458.6	0.035	10.1	60	454.8	60.3	454.9
D1	473.0	0.048	8.2	45	469.1	45.0	469.1
D2	488.9	0.036	11.1	47	474.5	53.8	481.4
D3	490.2	0.046	8.7	45	485.1	43.6	484.1
D4	567.9	0.025	15.6	49	513.9	56.1	534.3
D5	507.8	0.041	8.9	56	505.1	44.3	497.9
D6	594.5	0.030	13.1	50	566.9	47.2	560.0
E1	555.6	0.029	14.2	48	519.7	52.0	529.3
E2	526.2	0.041	9.9	44	514.7	42.6	512.7
E3	493.1	0.041	9.6	48	486.5	47.2	485.8
E4	715.0	0.024	17.3	46	613.5	51.9	644.2
E5	580.9	0.033	11.8	57	571.4	44.6	552.2
E6	559.4	0.040	9.5	43	545.5	39.6	539.3
F1	638.5	0.037	11.8	41	604.4	40.6	603.1
F2	1034.4	0.025	19.2	46	879.0	47.9	896.7
F3	893.4	0.028	16.8	46	799.7	45.8	798.1
F4	994.7	0.027	17.4	47	884.7	45.7	874.7
F5	891.2	0.026	16.2	48	805.2	46.0	792.6
F6	799.9	0.033	12.9	46	761.1	40.4	736.4

Appendix 2. Growth trajectories of 29 *Rana ornativentris* individuals in the rearing experiment and following expected terrestrial growth. IDs are shown on the upper side of the graph. Open circles show the actual growth data. Closed circles show the actual metamorphosis point. Stars show the optimal metamorphosis point calculated from the obtained model. Solid lines show Gompertz growth curves fitted to the growth data, and dotted lines show expected exponential terrestrial growth curves.

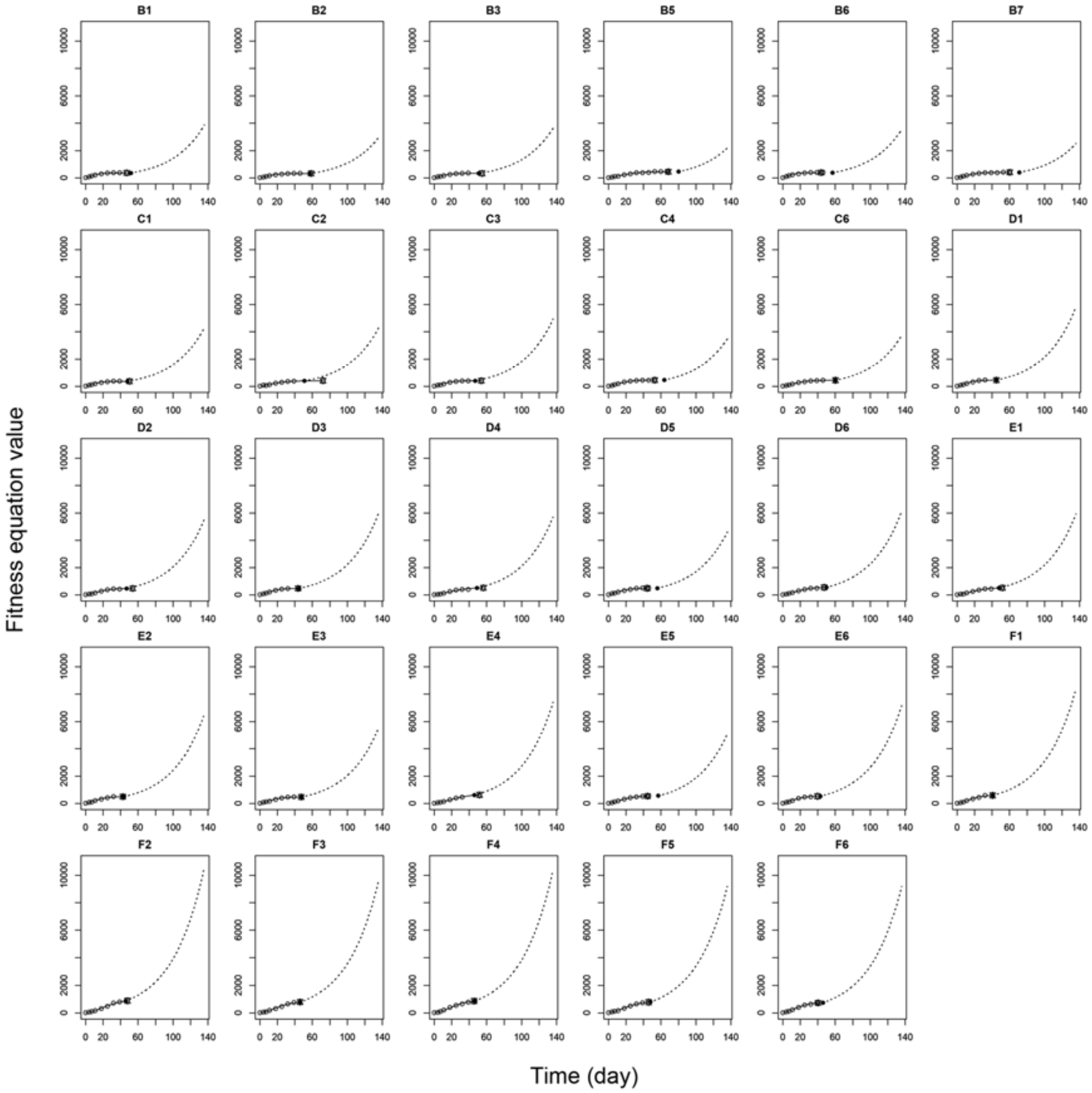
B1
B2
B3
B5
B6
B7
C1
C2
C3
C4
C6
D1
D2
D3
D4
D5
D6
E1
E2
E3
E4
E5
E6
F1
F2
F3
F4
F5
F6
Fitness equation value
Time (day)
